# Molecular and signaling mechanisms involved in cellular responses to mitochondrial DNA damage

**DOI:** 10.1590/1678-4685-GMB-2026-0020

**Published:** 2026-11-02

**Authors:** Sofia Ligia Guimarães-Ramos, Beatriz Kopel, Felippe Truglio Machado, Laís Yoshie Morikawa Muta, Rebeca Bueno-Alves, Wesley Roger Rodrigues Ferreira, Nadja Cristhina de Souza-Pinto

**Affiliations:** 1 Universidade de São Paulo, Instituto de Química, Departamento de Bioquímica, São Paulo, SP Brazil.; 2 Universidade Federal de Minas Gerais, Instituto de Ciências Biológicas, Departamento de Bioquímica e Imunologia, Belo Horizonte, MG, Brazil.

**Keywords:** Mitochondria, mitochondrial DNA, DNA damage, base excision, DNA damage tolerance

## Abstract

Mitochondria are essential organelles in mammalian cells, performing multiple functions ranging from energy metabolism and intracellular signaling to the regulation of cell fate. Proper mitochondrial function depends on the coordinated expression of two distinct genomes: the nuclear genome and the mitochondrial genome, known as mitochondrial DNA (mtDNA). Although most mitochondrial proteins are encoded by nuclear genes, mtDNA encodes 13 polypeptides that are essential components of the oxidative phosphorylation (OXPHOS) system and ATP production. For reasons that are not yet fully understood, mtDNA accumulates DNA damage at levels estimated to be 1-5 times higher than those observed in nuclear DNA, underscoring the importance of efficient DNA repair and damage tolerance mechanisms within mitochondria. Mammalian mitochondria are competent in several DNA repair pathways, with base excision repair being the best characterized. However, the molecular mechanisms underlying DNA damage response (DDR) pathways activated by mtDNA lesions remain poorly defined. In this review, we briefly summarize the established DNA repair pathways operating in mammalian mitochondria, which have been extensively discussed elsewhere, and focus primarily on DDR components implicated in the response to mtDNA damage, as well as on the cellular consequences of mtDNA damage accumulation.

## Introduction

Mitochondria are essential organelles that perform a wide array of cellular functions. In addition to producing ATP through oxidative phosphorylation, they play critical roles in calcium homeostasis, regulation of programmed cell death (apoptosis), biosynthesis of fatty acids, amino acids, and heme, thermogenesis, and intracellular signaling (reviewed in Nunnari and Suomalainen, 2012).

Owing to their endosymbiotic origin, mitochondria retain their own circular DNA (mtDNA), which is maternally inherited and present in hundreds to thousands of copies per cell, in contrast to the diploid and linear nuclear DNA (nDNA). Over more than two billion years of evolution, most of the ancestral α-proteobacterial genome was either lost or transferred to the nuclear genome (reviewed in Kleine et al., 2009). Consequently, the mitochondrial proteome, comprising more than 1,500 proteins, is predominantly encoded by nuclear genes, including all components of the molecular machinery required for mtDNA replication, transcription, translation, and repair (Scharfe et al., 2000).

The human mitochondrial genome is approximately 16.5 kb in length and encodes 13 polypeptides that constitute essential subunits of four of the five OXPHOS complexes, as well as 22 tRNAs and 2 rRNAs required for mitochondrial protein synthesis. Mitochondrial DNA replication and transcription are tightly regulated processes that ensure the maintenance of adequate mtDNA copy number and the coordinated expression of respiratory chain components in accordance with cellular energy demands (D’Souza et al., 2007; Falkenberg et al., 2024).

Although mtDNA is devoid of histones, which compact nuclear DNA (nDNA) into nucleosomes, it is organized into a nucleoprotein complex known as the mitochondrial nucleoid. While the overall structure of the nucleoid has been relatively well characterized, there is limited consensus regarding its precise composition, with some studies reporting up to 50 associated protein components (reviewed in Lee and Han, 2017). More recently, García-Villegas and colleagues identified 12 core nucleoid proteins in vivo in mice, all of which are components of the replication and transcription machinery or are involved in mitoribosome assembly (García-Villegas *et al*., 2025). Nevertheless, the principal structural component of the nucleoid is mitochondrial transcription factor A (TFAM), which binds mtDNA in a largely sequence-independent manner and coats it along its entire length. TFAM binding induces a sharp U-turn in the DNA, and subsequent TFAM dimerization and multimerization promote a high degree of compaction. By physically restricting access to the DNA, TFAM binding and nucleoid organization provide partial protection of the mitochondrial genome from damage; however, this compaction may also limit the accessibility of DNA repair machinery (Canugovi et al., 2010).

In the following sections, we discuss how cells may employ distinct and complementary strategies to detect and respond to different forms of mtDNA damage, examining the contributions of DNA repair pathways, degradation mechanisms, mitophagy, and mitochondrial dynamics. We further highlight broader cellular consequences, including effects on immune signaling and nuclear genome stability, and address major open questions and ongoing controversies in the field.

## DNA damage detection and repair in mitochondria

### mtDNA damage accumulation and detection

Like its nuclear counterpart, mtDNA is vulnerable to both endogenous and exogenous insults. However, likely owing to its proximity to the electron transport chain, where electron leakage generates reactive oxygen species (ROS) such as superoxide anions (O_2_^.-^) and hydrogen peroxide (H_2_O_2_), mtDNA appears to be particularly susceptible to oxidative damage. In addition, the lipid-rich environment of the inner mitochondrial membrane and the negative membrane potential across the inner membrane, which drives the accumulation of lipophilic positively charged molecules within the matrix, may further exacerbate mtDNA vulnerability to DNA lesions (reviewed in Muftuoglu et al., 2014).

Non-bulky lesions, such as small base modifications and abasic sites, represent the most common forms of DNA damage in mammalian cells. It has been estimated that up to 10,000 purine bases are lost per cell per day because of spontaneous depurination (Lindahl and Nyberg, 1972; Lindahl, 1993). In mitochondria, the high levels of oxidants generated by oxidative phosphorylation (OXPHOS) contribute to an even greater burden of base damage. Consequently, the mitochondrial genome is highly dependent on the base excision repair (BER) pathway to maintain its integrity.

Endogenous and exogenous agents can also induce bulky DNA lesions, which distort the DNA double helix and interfere with essential processes such as replication and transcription. Among exogenous sources, ultraviolet (UV) radiation is a major inducer of bulky lesions, generating cyclobutane pyrimidine dimers (CPDs) and 6,4-photoproducts (6,4-PPs) (Cadet et al., 2012). In normal human fibroblasts and embryonic kidney 293 cells, mtDNA exhibits similar levels of CPDs but higher levels of 6,4-PPs compared to nuclear DNA immediately after irradiation (Pascucci et al., 1997).

Chemical genotoxins can also induce bulky lesions in mitochondria. The chemotherapeutic agent cisplatin, for example, preferentially accumulates in the mitochondrial matrix (Dzamitika et al., 2006). In head and neck squamous cell carcinoma cell lines, platinum-DNA adducts were detected at levels approximately 300-fold higher in mtDNA than in nDNA, suggesting preferential adduct formation in the mitochondrial genome (Yang et al., 2006). Another genotoxic agent, formaldehyde (FA), is both an endogenous metabolic byproduct and an environmental contaminant (Dorokhov et al., 2015). FA can induce a broad spectrum of DNA lesions, including monoadducts, DNA-DNA crosslinks, DNA-protein crosslinks, and DNA-glutathione crosslinks (Yu et al., 2015). In primary foreskin fibroblasts, FA exposure altered mitochondrial gene expression, reduced mitochondrial membrane potential (ΔΨm), triggered PINK1-Parkin-dependent mitophagy, and induced double-strand breaks (DSBs) (Nadalutti et al., 2020; Nadalutti *et al*., 2021). The authors proposed that these DSBs likely result from replication fork collapse at unrepaired FA-induced lesions.

Similarly, the aldehyde acrolein-generated during lipid peroxidation and present as an air pollutant-induces mtDNA damage in a dose-dependent manner and preferentially affects mtDNA over nDNA in MRC-5 and A549 cells (Hruszkewycz, 1988; Wang et al., 2017). Evidence also supports the preferential formation of the DNA adduct N1-methyldeoxyguanosine (M1dG), produced by malondialdehyde or base propenals during lipid peroxidation, in mtDNA. M1dG accumulates in mtDNA at approximately twice the levels observed in nDNA and interferes with mitochondrial gene expression by inhibiting the mitochondrial RNA polymerase (POLRMT) activity in a promoter-dependent manner (Jeong, 2005; Cline et al., 2010).

Together, these findings support the idea that mtDNA accumulates substantial damage from both endogenous and exogenous sources. Mechanisms that repair, tolerate, or eliminate damaged mitochondrial genomes are therefore essential to preserve mtDNA stability and, consequently, mitochondrial function and cellular homeostasis. Figure 1 summarizes the known DNA repair pathways in mitochondria and the general consequences of unrepaired lesions.

**Figure 1 -:**
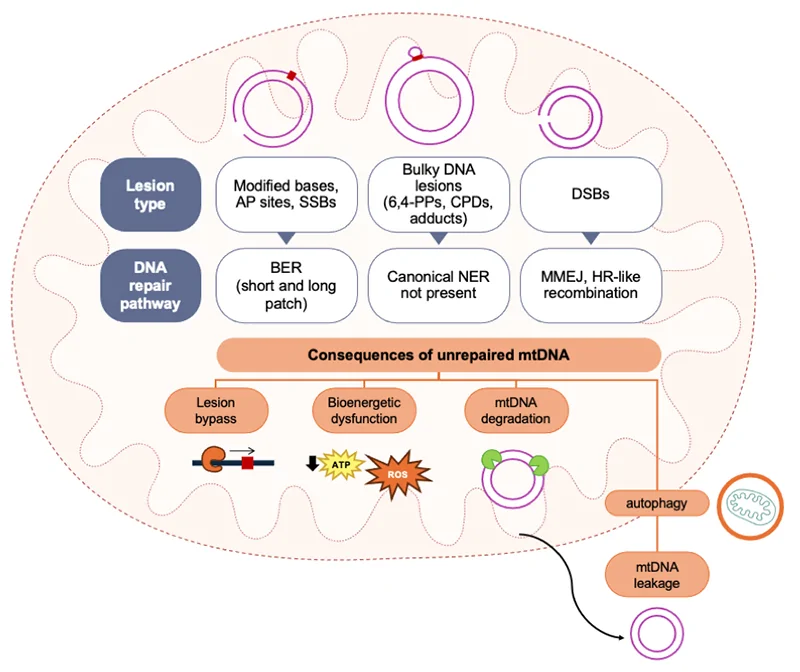
DNA damage repair and tolerance mechanisms in mammalian mitochondria. Depending on the lesion type, different DNA repair pathways will be recruited to deal with the damage. Inside the mitochondria, the figure depicts mitochondrial consequences to unrepaired mtDNA, such as lesion bypass by DNA damage tolerance mechanisms, bioenergetic dysfunction, and mtDNA degradation. Outside, the consequences of unrepaired mtDNA damage are shown, namely mtDNA leakage and degradation of heavily damaged mitochondria via mitophagy.

### The mitochondrial base excision repair - BER

Small DNA lesions, including single-strand breaks, oxidized and alkylated bases, and abasic (AP) sites, are primarily repaired through the BER pathway (Figure 1). BER proceeds through four major steps: (1) recognition and removal of the damaged base, (2) incision at the resulting AP site and processing of the 5′ and 3′ termini, (3) gap filling by a DNA polymerase, and (4) ligation of the DNA backbone. The pathway can be reconstituted *in vitro* with as few as four core enzymes (Dianov and Lindahl, 1994). BER was the first DNA repair pathway identified in mitochondria (Pettepher et al., 1991) and remains the best characterized repair mechanism in these organelles (reviewed in Alencar et al., 2019).

The initial step of BER is catalyzed by DNA glycosylases, which recognize and excise damaged bases by cleaving the N-glycosidic bond between the base and the deoxyribose sugar, thereby generating an AP site. Mammals possess 11 DNA glycosylases, each with defined but often overlapping substrate specificities (reviewed in Jacobs and Schär, 2012). Mechanistically, most glycosylases operate through a common strategy: they scan DNA for lesions, flip the damaged base out of the double helix, and insert it into a catalytic pocket for excision (Stivers et al., 1999).

Mitochondria are particularly active in repairing oxidative DNA damage and contain at least five glycosylases capable of removing oxidized bases. One of the most prevalent oxidative lesions, 8-oxoguanine (8-oxoG), can give rise to G:C→T:A transversions if left unrepaired (reviewed in Lindahl and Barnes, 2000). Oxoguanine DNA glycosylase (OGG1) is primarily responsible for excising 8-oxoG, especially from 8-oxoG:C base pairs. A mitochondrial isoform of OGG1 has been identified (Nishioka et al., 1999; Souza-Pinto et al., 2001), and its role in mtDNA maintenance has been extensively documented (Lia et al., 2018; reviewed in Alencar et al., 2019). Nonetheless, data in both flies (Itsara et al., 2014) and mice (Kauppila et al., 2018), and recently in a large human cohort (Gupta et al., 2026) indicate that most mutations that accumulate with age in mtDNA do not arise from oxidized bases, but rather from replication errors.

Additionally, an alternatively spliced isoform of the human MutY homolog (MYH), which removes adenines misincorporated opposite 8-oxoG, localizes to mitochondria (Ohtsubo et al., 2000; Parker et al., 2000). Germline mutations in MYH are associated with colorectal cancer predisposition (Al-Tassan et al., 2002), and emerging evidence implicates mtDNA alterations in colorectal cancer pathogenesis and clinical outcomes (reviewed in Koo et al., 2025).

Other oxidized bases, including formamidopyrimidines (FaPyA and FaPyG), thymine glycol (Tg), and 5-hydroxycytosine (5-hC), are substrates of NEIL and NTH family glycosylases. NEIL1, NEIL2, and NTHL1 have been detected in mammalian mitochondria (Takao et al., 1998; Hu et al., 2005; Mandal et al., 2012). In contrast, fewer glycosylases are known to repair deaminated or alkylated bases in mitochondria. UNG1, the mitochondrial isoform of uracil-DNA glycosylase (UDG), removes uracil arising from cytosine deamination, thereby preventing C:G→T:A transitions (Slupphaug et al., 1993; Nilsen et al., 1997). Moreover, alkyladenine DNA glycosylase (AAG), which excises certain alkylated bases, has been detected in human mitochondria, where it interacts with the mitochondrial single-stranded DNA-binding protein (mtSSB) (Van Loon & Samson, 2013).

Following base excision, an AP site is generated, which is itself a cytotoxic and potentially mutagenic lesion. Some DNA glycosylases, including OGG1, NEIL1, and NEIL2, possess intrinsic AP lyase activity (i.e., they are bifunctional glycosylases) and can directly cleave the DNA backbone at the AP site. In that case, the resulting strand break may require processing both at the 5’ and/or the 3’ ends, which can be accomplished by the apurinic/apyrimidinic endonuclease 1 (APE1), or by polynucleotide kinase/phosphatse (PNPK) (Tahbaz et al., 2012). In contrast, monofunctional glycosylases rely on APE1 to incise the phosphodiester backbone. In addition to its endonuclease activity, APE1 can function as a lesion sensor, bending the DNA and flipping the AP site into its catalytic pocket (Mol et al., 2000; Weaver et al., 2022). APE1 has been localized to mitochondria (Tell et al., 2005), where it participates not only in mtDNA repair but also in the processing of abasic sites in mitochondrial RNA (Barchiesi et al., 2021). 

Both short-patch and long-patch BER sub pathways operate in mitochondria (reviewed in Alencar et al., 2019). In short-patch BER, DNA polymerase γ (Polγ) inserts a single nucleotide to fill the gap (Stierum et al., 1999). In long-patch BER, Polγ incorporates 2-10 nucleotides, displacing the downstream strand and generating a flap structure that is removed by flap endonuclease 1 (FEN1) (Liu et al., 2008; Szczesny et al., 2008). More recently, DNA polymerase β (Polβ) has also been localized in mammalian mitochondria, and its role in mtBER has been demonstrated by two independent groups (Prasad et al., 2017; Sykora et al., 2017). Mitochondrial Polβ supports mtBER both *in vitro* and in cellular extracts and its strand displacement activity is stimulated by the mitochondrial helicase Twinkle (Baptiste et al., 2021), supporting a physiological role in maintaining mtDNA stability.

The remaining nick, bearing a 3′-OH and 5′-phosphate, is sealed by DNA ligase III (LIG3). LIG3 is the primary DNA ligase in mitochondria, and its loss results in embryonic lethality in mice (Puebla-Osorio et al., 2006), a phenotype largely attributed to mitochondrial dysfunction rather than nuclear defects. Consistently, mitochondrial LIG3 activity is essential for mtDNA maintenance, and its inactivation leads to severe mitochondrial impairment (Gao et al., 2011; Simsek et al., 2011).

Like other proteins involved in mtDNA maintenance, mtBER components are encoded by the nuclear genome and imported into the mitochondrial matrix. Most contain canonical mitochondrial targeting sequences (Pfanner, 2000), although some repair proteins detected in mitochondria lack obvious targeting signals and are presumed to be imported through alternative mechanisms. Thus, mtDNA stability also depends on the efficient recruitment and import of DNA repair proteins. Notably, Szczesny et al. (2003) demonstrated that the mitochondrial import of OGG1 and UDG is reduced in aged compared to young mice, as well as in senescent versus proliferating human fibroblasts, suggesting that impaired protein import may contribute to the age-associated accumulation of mtDNA lesions. Exercise has also been shown to modulate mtBER import, as physical activity increased OGG1 import into the mitochondrial matrix in rat skeletal muscle (Radak et al., 2009). In addition, mtDNA content appears to influence the recruitment of repair proteins: human ρ0 cells lacking mtDNA display reduced mitochondrial APE1 levels (Stuart et al., 2004), and APE1 mitochondrial translocation can be modulated by oxidative stress (Frossi et al., 2002; Barchiesi et al., 2020).

It is important to point out that because many DNA repair proteins are highly abundant in the nucleus and cytoplasm, demonstrating their genuine mitochondrial localization requires multiple complementary approaches. Using a single method, such as immunofluorescence microscopy or mitochondrial fractionation alone, may be insufficient due to the limited resolution of light microscopy and the frequent contamination of mitochondrial preparations with nuclear or endoplasmic reticulum components. Instead, convincing evidence should combine biochemical fractionation with protease protection assays, high-resolution microscopy, immunogold electron microscopy, proximity-labeling techniques, and functional assays demonstrating activity within mitochondria (for a proposed roadmap to refine the mitochondrial proteome, see Baker et al., 2024), particularly for proteins with low mitochondrial abundance or that translocate to mitochondria only under specific stress conditions, which is a common feature for DNA repair proteins, even in the nucleus (Alexeyev et al., 2013). Such rigorous validation is essential to distinguish bona fide mitochondrial proteins from experimental artifacts and to establish their physiological roles in mitochondrial DNA maintenance.

Recent evidence indicates that mitochondrial transcription factor A (TFAM) modulates BER in a lesion-, sequence-, and concentration-dependent manner. Optimal TFAM-to-DNA ratios enhance UNG1 and APE1 activities, whereas higher TFAM levels inhibit OGG1 and AAG and reduce the accumulation of repair intermediates (Urrutia et al., 2024). Furthermore, the presence of 8-oxodG strengthens TFAM binding at specific sequence motifs, providing a mechanistic explanation for OGG1 inhibition under these conditions. The fact that TFAM binding compacts DNA is also very relevant to mtDNA damaging processing and for mtDNA metabolism in general. We have shown earlier that TFAM binding limits access to DNA repair proteins (Canugovi et al., 2010) while recent work showed that the TFAM/DNA ratio, and consequently the nucleoid compaction, modulates mtDNA replication and transcription as well (Brüser et al., 2021). Collectively, these findings underscore that mtDNA repair efficiency is tightly regulated by TFAM abundance, DNA sequence context, and the nature of the lesion (Urrutia *et al*., 2024). 

Although PARP1 is classically regarded as a nuclear DNA damage sensor, accumulating evidence suggests that a fraction of mammalian PARP1 can localize to mitochondria, where it may participate in the maintenance of mitochondrial genome integrity and regulation of mitochondrial function. However, its mitochondrial localization remains controversial, as several studies have failed to detect significant constitutive mitochondrial PARP1, and the mechanisms governing its import are not fully resolved (reviewed in Herrmann and Yin, 2023). Current evidence suggests that mitochondrial PARP1 may be recruited under conditions of oxidative or genotoxic stress, where it interacts with components of the mitochondrial base excision repair machinery, including Polγ and Exonuclease G, thereby modulating mtDNA repair efficiency (reviewed in Brunyanszki et al., 2016). In addition, mitochondrial PARP1 has been implicated in the regulation of mitochondrial transcription, bioenergetics, NAD⁺ homeostasis, and cell death pathways through local PARylation reactions and crosstalk with nuclear PARP1 signaling (Lapucci et al., 2011). Consequently, the prevailing view is that mitochondrial PARP1 likely represents a regulated, context-dependent pool of the enzyme rather than a constitutively abundant mitochondrial protein, although its precise physiological importance remains an active area of investigation.

Although many aspects of mtBER have been elucidated, several key questions remain unresolved. All mtBER enzymes are nuclear-encoded and must be imported into mitochondria, yet the mechanisms governing their targeting specificity and import efficiency are not fully understood. Moreover, although mtBER activity has been associated with the mitochondrial membrane fraction (Stuart et al., 2005), it remains unclear how BER enzymes are distributed among the numerous mtDNA copies within a cell and how repair is coordinated with mitochondrial dynamics and mitophagy. Whether mtBER efficiency declines with aging or metabolic stress is also a matter of debate, as is the extent to which mtBER is regulated by cellular energy demand. Addressing these questions will be essential to fully understand how mitochondrial genome stability is maintained under physiological and pathological conditions.

### Repair of bulky lesions and DNA damage tolerance

Early studies of mtDNA repair reported an apparent absence of nucleotide excision repair (NER) activity, the canonical nuclear pathway responsible for the removal of bulky DNA lesions, which led to the misleading conclusion that mitochondria lacked DNA repair capacity altogether (Clayton et al., 1974). To date, no compelling evidence supports the existence of a functional NER pathway in mammalian mitochondria (Figure 1). However, in *Trypanosoma cruzi*, a transcription-coupled nucleotide excision repair-like mechanism dependent on Cockayne syndrome B protein (CSB) has been described following cisplatin and UV-C exposure (Repolês et al., 2025), raising the possibility that certain organisms may harbor modified forms of NER within mitochondria.

In human cells, several canonical NER factors, including Cockayne syndrome A (CSA) and B (CSB) proteins (Kamenisch et al., 2010), Xeroderma pigmentosum complementation group D (XPD) (Liu et al., 2015), Methyl Methanesulfonate Sensitivity gene 19 (MMS19) (Wu et al., 2018), and Rad23 homologue A (RAD23A) (Pohjoismäki et al., 2012), have been reported to localize to mitochondria. Although these proteins do not appear to function in a classical NER pathway within this organelle, they contribute to mtDNA stability through alternative mechanisms. CSB was first implicated in mitochondrial DNA maintenance when reduced repair of 8-oxoG was observed in the mtDNA of CSB-deficient patients and mice (Stevnsner et al., 2002). Subsequent studies demonstrated that CSA and CSB interact with mitochondrial BER components, including mtOGG1 and mitochondrial single-stranded DNA binding protein 1 (mtSSBP1) (Kamenisch *et al*., 2010), and can stimulate mitochondrial transcription. *In vitro* assays showed that CSB enhances POLRMT activity, whereas extracts from CSB-deficient cells display reduced mitochondrial gene transcription (Berquist et al., 2012). The diverse mitochondrial functions of CSB and their relevance to Cockayne syndrome pathology have been comprehensively reviewed elsewhere (Tiwari et al., 2021).

Given the apparent absence of canonical NER in mammalian mitochondria, alternative mechanisms must ensure mtDNA replication in the presence of unrepaired, replication-blocking lesions typically handled by NER in the nucleus. In the nuclear genome, DNA damage tolerance (DDT) pathways enable replication to proceed across damaged templates (Ler and Carty, 2022). DDT mechanisms are highly conserved among eukaryotes (Ulrich, 2007) and are essential for mammalian viability. Indeed, simultaneous loss of the translesion synthesis polymerase REV1 and mutation of the PCNA mono-ubiquitination site (K164), which regulates polymerase switching at stalled replication forks, results in embryonic lethality (Buoninfante et al., 2023).

In the nucleus, DNA damage tolerance (DDT) pathways can be broadly categorized according to their dependence on proliferating cell nuclear antigen (PCNA) ubiquitination. Translesion synthesis (TLS) and homology-directed mechanisms require PCNA ubiquitination, whereas repriming appears to be independent of PCNA mono-ubiquitination. TLS involves specialized DNA polymerases, primarily members of the Y family, that bind mono-ubiquitinated PCNA and transiently replace the stalled replicative polymerase to insert nucleotides opposite the lesion (Bienko et al., 2005; Hoitsma et al., 2020). Certain lesions require a two-step TLS process, in which an “inserter” polymerase incorporates a nucleotide opposite the damaged base, followed by an “extender” polymerase, typically DNA polymerase ζ, before PCNA is deubiquitinated and replicative synthesis resumes (Prakash et al., 2005).

Homology-directed DDT pathways include fork reversal (FR) and template switching (TS), both considered largely error-free and triggered by PCNA poly-ubiquitination (Xu et al., 2015). During fork reversal, replisome uncoupling promotes remodeling of the three-way replication fork into a four-way junction, facilitating lesion bypass or repair through recombination-mediated mechanisms (Zellweger et al., 2015; Berti et al., 2020; Kavlashvili et al., 2023; Adolph and Cortez, 2024). In template switching, a RAD51-ssDNA nucleofilament invades the newly synthesized sister chromatid to use it as a damage-free template, enabling DNA polymerase δ-mediated synthesis followed by resolution of the recombination intermediate (Ler and Carty, 2022).

In addition to these PCNA-dependent mechanisms, a third DDT pathway, repriming, allows replication to restart downstream of a lesion. This process depends on the primase-polymerase PrimPol, identified in the early 2010s, which can synthesize both ribonucleotide (NTP) and deoxyribonucleotide (dNTP) primers and perform limited TLS across lesions such as abasic sites, 8-oxoG, cyclobutane pyrimidine dimers (CPDs), and 6,4-photoproducts (6,4-PPs) (García-Gómez et al., 2013; Bianchi et al., 2013). Repriming leaves behind a single-stranded DNA gap that can subsequently be filled by TLS polymerases (Mourón et al., 2013; Jahjah et al., 2024).

Although mitochondrial DDT mechanisms remain incompletely understood, current evidence suggests that damage tolerance in mammalian mitochondria primarily involves TLS and/or repriming. Homology-based mechanisms such as template switching might occur, as the mitochondrial helicase TWINKLE exhibits homologous strand-exchange activity (Sen et al., 2016); however, canonical nuclear TS factors have not been identified in mitochondria.

There is substantial evidence that the replicative mitochondrial DNA polymerase, DNA polymerase γ (Polγ), can bypass a variety of DNA lesions, including CPDs (Park et al., 2022), benzo[a]pyrene and benzo[c]phenanthrene diol epoxides (Graziewicz et al., 2004), 8-oxoG (Graziewicz *et al*., 2007), the acrolein-induced adduct dHOPdG (Kasiviswanathan et al., 2013), and platinum-DNA adducts (Vaisman et al., 1999). Other TLS-related polymerases, including REV1 (Fakouri et al., 2017; Anugula et al., 2023), DNA polymerase θ (Wisnovsky et al., 2018), DNA polymerase ζ (Singh et al., 2015), and PrimPol (García-Gómez et al., 2013), have also been detected in mitochondria. However, there is limited in vivo evidence supporting their functional involvement in mitochondrial TLS. In fact, in *in vitro* experiments, PrimPol did not promote replication fork bypass in a 8-oxodG-containing substrate (Stojkovič et al., 2016). The molecular basis of TLS activation and polymerase recruitment in mitochondria remains unclear, particularly because PCNA is absent and no clamp-like functional analog has been identified (Falkenberg, 2018). Given its scaffolding role in nuclear TLS, REV1 has been proposed as a potential mediator of polymerase switching in mitochondria, at least in yeast (Gilea et al., 2023).

Repriming may also contribute to mtDNA maintenance. Several regulators implicated in nuclear DDT, including PolDIP2 (Guilliam et al., 2016; Hernandes et al., 2017; Tsuda et al., 2019) and p53 (Castaño et al., 2024), localize to mitochondria and associate with nucleoids. Although their participation in a defined mitochondrial DDT pathway has not yet been demonstrated, their presence suggests that conserved or adapted tolerance mechanisms may operate within the organelle. Notably, PrimPol can reinitiate stalled mtDNA replication after DNA damage, and reprime mtDNA synthesis from nonconventional synthesis (Torregrosa-Muñumer et al., 2017, Bailey et al., 2019). Thus, despite the absence of canonical repair systems such as NER, mitochondria may rely on specialized damage-tolerance strategies-particularly TLS and repriming-to sustain mtDNA replication and preserve genome integrity under genotoxic stress.

### Double strand breaks: Repair or degradation?

Double-strand breaks (DSBs) are among the most cytotoxic forms of DNA damage, as they disrupt the continuity of the genetic material and eliminate the intact complementary strand required for accurate repair. DSBs can arise from both exogenous and endogenous sources, including ionizing radiation, oxidative stress, crosslinking agents, and stalled replication forks (reviewed in Giglia-Mari et al., 2011). In the nuclear genome, DSBs are primarily repaired through homologous recombination (HR) or non-homologous end joining (NHEJ). HR requires a sister chromatid as a template and therefore predominates during the S and G2 phases of the cell cycle (Kadyk and Hartwell, 1992), whereas NHEJ operates throughout the remaining phases, when sister chromatids are not available (Wyman and Kanaar, 2006).

Although the nuclear DSB repair machinery is well characterized, mitochondrial DSB repair remains less well understood. Several core components of classical NHEJ, including Ku70 and Artemis, have not been conclusively detected in mitochondria (Li et al., 2007; reviewed in Alencar et al., 2019). However, a mitochondrial specific alternative 68-kD isoform of Ku80, lacking a C-terminal domain of the full length 80-kD isoform, has been identified (Coffey & Campbell, 2000). This isoform retains DNA binding activity, raising the possibility that a non-canonical NHEJ-like pathway may operate in this organelle (Figure 2). Consistent with this hypothesis, mitochondrial extracts from human tissues exhibit microhomology-mediated end joining (MMEJ) activity involving FEN1, DNA ligase III, MRE11, and PARP1 (Tadi et al., 2016). Among these factors, PARP1 has emerged as a potential mitochondrial DNA damage sensor, displaying higher enzymatic activity in mitochondria than in the nucleus (Du et al., 2003), and its depletion compromises mtDNA integrity (Rossi et al., 2009). Moreover, nuclear PARP1 regulates the expression of genes involved in mtDNA repair and transcription, suggesting functional crosstalk between nuclear and mitochondrial DNA damage responses (Lapucci et al., 2011).

**Figure 2 -:**
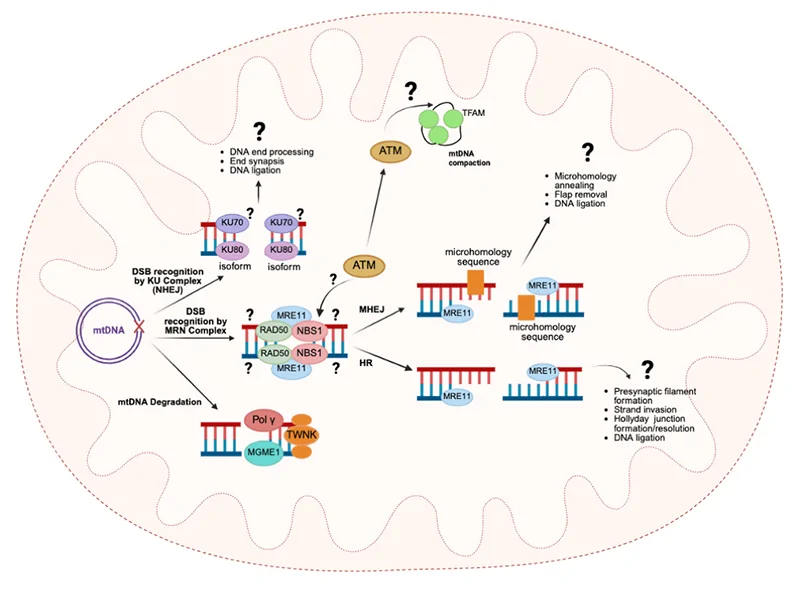
Recognition and processing of double strand breaks in mtDNA. Proposed mechanisms for the recognition, repair, and degradation of mtDNA DSBs are shown. Evidence supports the presence of a mitochondrial Ku80 isoform with DNA end-binding activity, whereas the occurrence and functional relevance of Ku70 and downstream components of the canonical non-homologous end joining (NHEJ) pathway remain uncertain. Components of the MRN complex have also been detected in mitochondria, with MRE11 showing the strongest experimental support, while the mitochondrial localization and functions of RAD50 and NBS1 remain less well established. Current data suggest that MRN-dependent processing may contribute to microhomology-mediated end joining (MMEJ) and homologous recombination-like (HR-like) mechanisms; however, the downstream repair steps, including DNA end processing, strand invasion, Holliday junction formation, and DNA ligation, have not been conclusively demonstrated in mammalian mitochondria. ATM may participate in mtDNA damage signaling or nucleoid remodeling, potentially through interactions with TFAM, although its precise role remains unclear. In addition to repair pathways, damaged linear mtDNA molecules may be eliminated through degradation mechanisms involving MGME1, DNA Polγ, and the TWNK helicase. Question marks indicate processes or molecular components that remain incompletely characterized or experimentally unresolved. Figure created with BioRender.com.

In addition to NHEJ-like mechanisms, accumulating evidence supports the existence of HR-like processes in mitochondria. Although mitochondria lack canonical sister chromatids, the presence of multiple circular mtDNA copies may provide homologous templates for recombination. The MRN complex (MRE11-RAD50-NBS1), which recognizes and resects DSBs during nuclear HR initiation, has been detected in mitochondria (Dmitrieva et al., 2011; Tadi et al., 2016; Dahal et al., 2018; Li et al., 2019; Qi et al., 2020). Depletion of MRE11 or NBS1 reduces mitochondrial recombination activity, and MRE11 binds directly to mtDNA, particularly following oxidative or radiomimetic stress, suggesting a role in DSB processing and stabilization (Dmitrieva *et al*., 2011; Li *et al*., 2019). RAD50 and NBS1 have also been localized to mitochondria, although their specific functions in mtDNA maintenance remain less clearly defined (Dahal *et al*., 2018). Together, these findings support the possibility that mitochondria employ HR-like mechanisms, although the complete pathway and its regulatory framework remain to be elucidated (Figure 2).

In addition to MRN, ATM, a central kinase in the nuclear DNA damage response, is also present in mitochondria (Valentin-Vega et al., 2012; Blignaut et al., 2019). ATM-deficient cells display impaired electron transport chain function, reduced ATP production, increased oxidant production, and decreased levels of DNA ligase III, resulting in the accumulation of damaged mtDNA (Sharma et al., 2014; Shimura et al., 2016; Blignaut *et al*., 2019). Although ATM can be activated by mitochondrial oxidants, its direct role in sensing or signaling mtDNA DSBs remains unclear. An important open question is whether ATM modifies mitochondrial DNA-binding proteins, such as TFAM, to regulate mtDNA accessibility, analogous to its phosphorylation of H2AX in the nucleus (Figure 2). The presence of MRN and ATM in mitochondria therefore suggests partial conservation of DSB recognition machinery, but their interactions and downstream signaling pathways require further clarification.

Beyond repair, degradation of linearized mtDNA represents an alternative mechanism for maintaining mitochondrial genome integrity (Figure 2). Because mtDNA is present in multiple copies, DSBs frequently result in the selective elimination of linearized molecules rather than their repair. Exonuclease-mediated degradation involves MGME1, the exonuclease activity of DNA polymerase γ (Polγ), and the TWNK helicase (Peeva et al., 2018; Trombly et al., 2023) and appears to be mechanistically distinct from autophagy or mitophagy. Knockdown of other mitochondrial nucleases-including EndoG, ExoG, DNA2, APEX2, MRE11, RBBP8, or FEN1-does not impair clearance of linear mtDNA (Moretton et al., 2017). Conversely, defective degradation can promote the accumulation of rearranged or deleted mtDNA molecules, often associated with DNA ligase III-dependent NHEJ-like events (Nicholls et al., 2014; Phillips et al., 2017; Peeva *et al*., 2018). These observations highlight a dynamic balance between repair and degradation in preserving mitochondrial genome stability under genotoxic stress.

Overall, although mitochondria share selected components of nuclear DSB repair pathways-such as MRN and ATM-the underlying mechanisms remain incompletely understood (Figure 2). Current evidence supports a combination of microhomology-mediated end joining, HR-like recombination between mtDNA copies, and regulated degradation of linearized genomes as central strategies for maintaining mtDNA integrity. Future studies employing CRISPR-based approaches to delete potential mitochondrial DSBR proteins, long-read sequencing, and high-resolution imaging will be critical for elucidating the precise dynamics and regulatory architecture of mitochondrial DSB repair and degradation (Trombly et al., 2023).

## Cellular consequences of mtDNA instability

### Bioenergetic consequences

Mitochondrial DNA (mtDNA) is essential for proper OXPHOS because it encodes 13 core subunits of the electron transport chain (ETC) complexes I, III, IV, and the ATP synthase (Complex V), as well as all the RNAs necessary for mitochondrial protein synthesis. Damage to mtDNA, whether from oxidative stress, replication errors, or environmental insults, can have profound effects on cellular energy metabolism (Wallace, 2012).

MtDNA damage frequently results in decreased expression of ETC subunits, which compromises electron transport and diminishes the proton gradient across the inner mitochondrial membrane. Dysfunctional complexes I and III are especially prone to mtDNA mutation burden, leading to reduced ATP synthesis and lower cellular energy availability (Bratic and Trifunovic, 2010). This reduction in ATP is a hallmark of many age-associated and mitochondrial diseases, and affects, preferentially, high energy-demanding tissues such as brain, heart, and skeletal muscle, which become energetically compromised (Schon and Manfredi, 2003). In addition, when OXPHOS is compromised, cells may shift toward glycolysis (“Warburg-like” metabolic reprogramming) even in normoxia, leading to altered metabolic fluxes and lactate accumulation (Nickel et al., 2020). While this can partially sustain ATP levels in the short term, it is less efficient and can contribute to pathology, particularly in tissues that rely heavily on oxidative metabolism. Figure 3 depicts a scheme of the bioenergetic and signaling consequences of accumulation and mtDNA.

**Figure 3 -:**
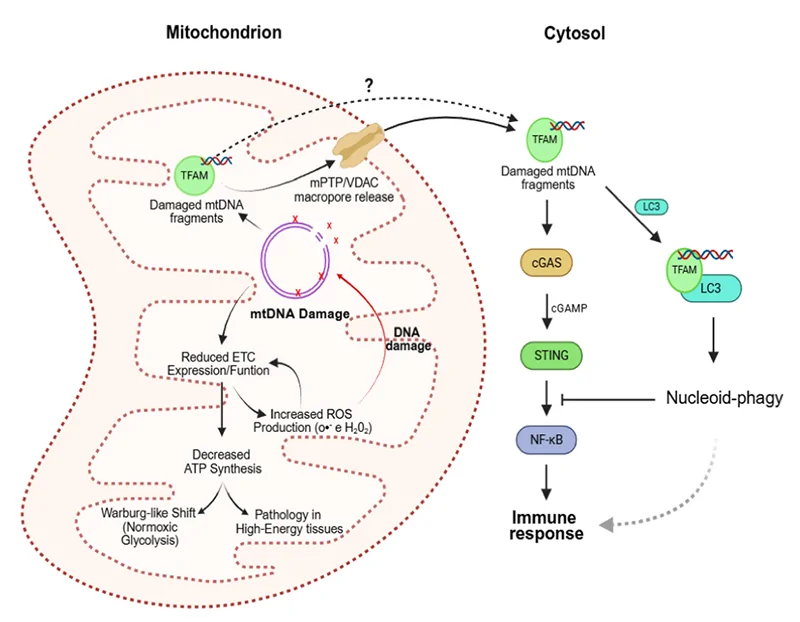
Signaling responses activated by mtDNA damage. The accumulation of mtDNA damage generates a deleterious cycle as it reduces the expression and function of subunits of the ETC, decreasing ATP synthesis and inducing a metabolic shift toward normoxic glycolysis (Warburg-like shift). Concomitantly, ETC dysfunction increases production of O•- and H2O2, which feed back into and amplify mtDNA damage. In addition, TFAM-bound mtDNA fragments may be released into the cytosol through macropores formed by the mitochondrial permeability transition pore (mPTP) and the voltage-dependent anion channel (VDAC) - although the exact mechanisms mediating transit across mitochondrial membranes remains to be elucidated (represented by the question mark). In the cytosol (right), the released mtDNA follows two main pathways: 1) pro-inflammatory pathway (cGAS-STING), when free mtDNA is recognized by the cGAS sensor, which synthesizes cGAMP to activate the STING adapter protein, which subsequently recruits the transcription factor NF-κB, culminating in the activation of the innate immune response; 2) nucleoid-phagy pathway, when cytosolic TFAM binds directly to the autophagic protein LC3, targeting the damaged nucleoids for selective autophagic degradation. This pathway acts as a protective mechanism that attenuates inflammatory signaling, inhibiting excessive NF-κB activation and modulating the immune response. Figure created with BioRender.com.

Moreover, damaged mtDNA contributes to defective ETC function, which promotes electron leakage and increases production of oxidant species such as O_2_^.-^ and H_2_O_2_ (Kowaltowski et al., 2009). The resulting redox imbalance both damage additional mtDNA and oxidize lipids and proteins within mitochondria, creating a vicious cycle of mitochondrial dysfunction (Alexeyev et al., 2013). This oxidant-mediated feedback loop contributes not only to bioenergetic decline but also to cellular stress signaling and apoptosis.

Cells with significant mtDNA damage often activate compensatory mechanisms, such as increased mitochondrial biogenesis signaling through the transcriptional co-activator PGC-1α, in an attempt to restore energy balance (West et al., 2015), although chronic damage can overwhelm these responses. mtDNA damage also affects mitochondrial dynamics, skewing the balance toward fission over fusion, which can further reduce mitochondrial efficiency and amplify energetic deficits (Chen et al., 2010).

The bioenergetic consequences of mtDNA damage extend beyond mitochondria themselves and affect cellular homeostasis through reduced ATP output, widespread redox imbalance that contribute to inflammation, cell death, and impaired tissue regeneration (Lee and Wei, 2007). In neurodegenerative diseases such as Parkinson’s and Alzheimer’s, mtDNA damage correlates with reduced ETC activity and neural energy failure (Lin and Beal, 2006). Similarly, in heart failure and metabolic syndromes, mtDNA integrity is closely linked with contractile and metabolic dysfunction (Taylor and Turnbull, 2005).

### Signaling responses activated by cytosolic mtDNA

Due to their bacterial origin, mitochondria retain several prokaryotic features that are recognizable by the immune system, including their own DNA (mtDNA) (Fang et al., 2016; Kausar et al., 2020; De Gaetano et al., 2021). Extracellular mtDNA is classified as one of the damage-associated molecular patterns (DAMPs), capable of triggering inflammatory processes (Rock and Kono, 2008). The first receptor identified as capable of recognizing mtDNA was TLR9 (Hemmi et al., 2000; Bauer et al., 2001), whose activation induces an inflammatory response via mitogen-activated protein kinase (MAPK) (Zhang et al., 2010) and NF-κB (Amarante-Mendes et al., 2018). Evidence of mtDNA escape emerged in the 1990s from studies in *Saccharomyces cerevisiae*, demonstrating that mtDNA could migrate to the nucleus and that increased migration resulted in nuclear genomic instability, with some associated mutations directly linked to uncontrolled mtDNA release (Thorsness and Fox, 1990, 1993). Currently, major focus has been on the role of this release in innate immune signaling (Arnoult et al., 2009; Ohta and Nishiyama, 2011; Sprenger et al., 2021), and this phenomenon has been associated with various pathological conditions, including neurodegenerative processes (Mathew et al., 2012; Wilkins et al., 2015).

Recent studies demonstrate that mtDNA activates the immune response through the stimulator of interferon genes (STING) pathway (Figure 3), a central mediator in mtDNA sensing (Paludan and Bowie, 2013; Dib et al., 2015). STING is an adaptor that binds cyclic dinucleotides (CDNs) produced in mammalian cells by cGAS, which recognizes foreign DNA in the cytoplasm (Burdette et al., 2011; Civril et al., 2013; Lu et al., 2024). The cGAS-STING pathway is initiated when double-stranded DNA accumulates in the cytoplasm and is detected by cGAS, which in response produces 2′3′-cGAMP. This molecule binds to STING, leading to its activation (Wu et al., 2013). STING activation results in its dimerization and translocation, forming a complex with TANK-binding kinase 1 (TBK1), which leads to activation of nuclear factor kappa B (NF-κB) and the induction of inflammatory genes, particularly type I interferons (Uggenti et al., 2019). In addition to regulating inflammatory gene expression, this pathway also has transcription-independent functions, including the induction of different forms of cell death (Saitoh et al., 2009; Tang et al., 2016; Gaidt et al., 2017).

MtDNA has been reported as the main cytosolic DNA molecule required to activate the STING pathway, and mtDNA release due to damage accumulation has been proposed to potentiate this response (Kate et al., 2024). However, it remains unclear which stimuli favor its release. What is known so far is that during apoptosis, proteins such as BAX and BAK form macropore complexes that facilitate mtDNA release (McArthur et al., 2018). It has also been shown that induction of the mitochondrial permeability transition pore (mPTP) concomitant with oxidative lesions in mtDNA results in increased cytosolic release of small DNA fragments (smaller than 1000 bp) (García et al., 2005). Similarly, ionizing radiation in mice also led to increased cytoplasmic release of mtDNA via the mPTP (Patrushev et al., 2006). Another channel possibly involved in mtDNA release is the voltage-dependent anion channel (VDAC), whose oligomerization is stimulated by small mtDNA fragments (Kim et al., 2019). Recently, it was demonstrated that rapid calcium uptake drives mPTP opening and VDAC oligomerization, resulting in the release of oxidized mtDNA (Xian et al., 2022). Despite these findings, it is still unclear how mtDNA transits between the inner and outer mitochondrial membranes, what biological signal triggers activation of this process, and what the size of the released fragments is in the absence of mitochondrial rupture (Figure 3).

### Mito- and nucleoid-phagy in response to mtDNA damage

To date, there is no direct evidence that mtDNA damage per se activates mitophagy. Instead, the prevailing model proposes that the accumulation of mtDNA damage interferes with mtDNA replication and transcription, thereby compromising oxidative phosphorylation (OXPHOS). This dysfunction ultimately leads to decreased mitochondrial membrane potential (Δψm), redox imbalance, and proteotoxic stress-well-established triggers of PINK1-Parkin-dependent mitophagy (Youle and van der Bliek, 2012; Pickles et al., 2018). In this framework, mitophagy is activated indirectly as mtDNA damage accumulates and becomes fixed as mutations or deletions that impair the electron transport chain (Stewart and Chinnery, 2015). However, whether damaged mtDNA itself, prior to the onset of overt respiratory dysfunction, can trigger mitophagy remains an open question, particularly given that degradation of the entire organelle represents a drastic response compared to alternative mechanisms such as mtDNA repair or selective degradation pathways.

Recent evidence supports the existence of early, mtDNA-responsive mitophagy pathways. Shu et al. (2021) demonstrated that treatment with H₂O₂ or 3-NPA induces a PINK1-Parkin-independent, oxidative stress-driven form of mitophagy mediated by ATAD3B, suggesting that mtDNA or nucleoid instability may initiate mitophagy even before measurable bioenergetic decline. Similarly, Sen et al. (2022) showed that mitochondrial membrane proteins, together with VPS35, orchestrate the selective removal of damaged mtDNA, indicating the existence of mitophagy-related mechanisms that specifically target the mitochondrial genome.

A complementary mechanism to whole-organelle mitophagy is nucleoid-phagy, in which mtDNA nucleoids are selectively eliminated to preserve mitochondrial function while minimizing inflammatory signaling. Liu et al. (2024) reported that cytosolic TFAM can directly bind LC3, enabling selective, LC3-dependent autophagic degradation of damaged nucleoids without inducing complete mitochondrial turnover (Figure 3). This pathway likely functions as a first-line protective mechanism to prevent cytosolic mtDNA exposure and subsequent activation of innate immune sensors such as cGAS-STING. However, nucleoid disposal appears to be mechanistically diverse and, under certain stress conditions, may instead promote inflammation. Newman et al. (2024) demonstrated that mtDNA replication stress can reroute nucleoids into a pro-inflammatory endosomal degradation pathway distinct from LC3-dependent nucleoid-phagy. This endosomal route couples nucleoid clearance to innate immune activation, highlighting that nucleoids can be processed through parallel pathways that either suppress or potentiate inflammation depending on the nature of the stress and the molecular machinery engaged.

### Signaling responses to mtDNA instability

Although DNA breaks are not the most frequent lesions observed in mtDNA, most experimental studies linking mtDNA damage to signaling pathway activation point to mtDNA double-strand breaks (DSBs) as potent initiators of coordinated, multi-layered stress responses that extend well beyond the mitochondrion. These findings position mtDNA as a genotoxic sentinel capable of modulating nuclear and cytosolic physiology. Tigano et al. (2021) demonstrated that mtDNA breaks induce BAX/BAK-dependent permeabilization of the mitochondrial outer membrane, enabling the selective release of mitochondrial RNA species into the cytosol. There, these RNAs activate the RIG-I-MAVS pathway and trigger type I interferon signaling, establishing an antiviral-like transcriptional program that enhances immune surveillance and amplifies damage-responsive pathways.

In a distinct yet complementary mechanism, Fu et al. (2023) showed that mtDNA breaks activate the DELE1-HRI-eIF2α arm of the Integrated Stress Response (ISR) independently of mtDNA release. Activation of the ISR transiently reprograms cytosolic translation, promotes mitochondrial proteostasis, and supports the recovery of mtDNA copy number and respiratory capacity, thereby contributing to the preservation of mitochondrial homeostasis under genotoxic stress.

In addition, Wu et al. (2019) reported that both acute and chronic mtDNA stress activate the cGAS-STING pathway, resulting in sustained formation of a non-canonical, unphosphorylated ISGF3 complex and upregulation of interferon-stimulated genes, including PARP9. This transcriptional response enhances nuclear DNA (nDNA) repair efficiency, accelerating the resolution of double-strand breaks marked by γH2AX and 53BP1. Notably, the resulting increase in repair capacity confers resistance to nuclear genotoxic agents such as doxorubicin in both cultured cells and mouse models. These findings suggest that mtDNA-initiated signaling can directly influence chemotherapeutic responsiveness by priming the nucleus to better withstand therapy-induced DNA damage.

Together, these findings indicate that the cellular consequences of mtDNA breaks go beyond impairing mitochondrial function; they engage a coordinated network of immune, metabolic, and genome surveillance pathways, thereby positioning mtDNA as a key mediator of mitochondria-to-nucleus signaling and a genuine sensor of genotoxic stress. Through these interconnected signaling cascades, the integrity of mtDNA emerges as a dynamic determinant of cellular fate decisions, stress adaptation, and responses to therapeutic interventions.

### Nuclear insertion of mtDNA

During the evolution of the endosymbiotic event that gave rise to mitochondria, most of the ancestral bacterial genome was either lost or transferred to the nuclear genome, generating what are known as nuclear mitochondrial DNA segments (NUMTs). The human genome is estimated to contain between approximately 200 and 600 NUMTs, depending on the study (Lascaro et al., 2008). 

While these sequences were long considered merely evolutionary relics, recent evidence suggests that active transfer of mtDNA fragments to the nucleus still occurs and may have pathological consequences. Ju et al. (2015) reported frequent somatic mtDNA insertions into the nuclear genome in cancer samples, and a subsequent comprehensive analysis of more than 2,500 cancer genomes confirmed that such insertions are common in tumors (Yuan et al., 2020). 

Although the molecular mechanisms underlying these events remain unclear, recent findings indicate that de novo integration of mtDNA into nuclear chromosomes can be triggered by stress, such as environmental mutagens that induce double-strand breaks (DSBs) (Harutyunyan et al., 2020), supporting the notion that genomic instability facilitates the incorporation of mtDNA fragments into the nuclear genome (Harutyunyan, 2024). Furthermore, Wu et al. (2024) identified significant nuclear-mtDNA fusion events at Cas9 target sites, with increased frequencies when mitochondrial genome editing using mitoTALENs or the DdCBE base editor was performed. Importantly, co-expression of the exonucleases TREX1 or TREX2 during DdCBE-mediated editing reduced these events, suggesting that the availability of released mtDNA fragments is required for their integration.

### Functional interplay between mtDNA damage responses

As reviewed here, mitochondria have evolved multiple mechanisms to cope with DNA damage that extend beyond canonical DNA repair pathways, and accumulating evidence indicates that these responses function as an integrated quality control network rather than as independent processes. It seems clear that low levels of lesions are primarily processed through BER, while additional mechanisms such as single strand break repair and mismatch correction may contribute under specific circumstances (Alexeyev et al., 2013, Muftuoglu et al., 2014). However, when DNA damage exceeds the repair capacity of the organelle or involves complex lesions that cannot be efficiently repaired, mitochondria can selectively eliminate damaged DNA molecules through degradation mediated by nucleases such as MGME1, EXOG, and the mitochondrial replication machinery itself, thereby preventing the propagation of dysfunctional genomes (Peeva et al., 2018; Nissanka and Moraes, 2020). This balance between repair and degradation appears to be critical for maintaining heteroplasmy and preserving mitochondrial genome integrity without relying on extensive repair pathways analogous to those present in the nucleus.

Beyond local genome maintenance, mtDNA damage also initiates organelle-wide and cellular stress responses that coordinate mitochondrial quality control with cell fate decisions. Persistent mtDNA lesions can alter mitochondrial membrane potential and oxidative phosphorylation, promoting mitochondrial fission and the selective removal of dysfunctional organelles by PINK1/Parkin-dependent or independent mitophagy pathways while simultaneously activating retrograde signaling mechanisms, including the integrated stress response, mitochondrial unfolded protein response (UPRmt), and innate immune pathways triggered by the release of mtDNA into the cytosol (Suomalainen and Battersby, 2018; Tigano et al., 2021; Yu et al., 2024). These pathways influence nuclear gene expression, mitochondrial biogenesis, metabolism, and inflammatory responses, allowing cells to adapt to mitochondrial dysfunction or eliminate irreversibly damaged mitochondria. Rather than representing separate outcomes of mtDNA damage, DNA repair, mtDNA turnover, mitophagy, and stress signaling are increasingly recognized as interconnected layers of mitochondrial genome surveillance that, collectively, determine whether mitochondrial function is restored or damaged organelles are eliminated to preserve cellular homeostasis.

## Conclusions

Mitochondrial DNA is particularly vulnerable to damage accumulation and, in fact, accumulates more lesions than the nuclear genome, underscoring the importance of robust DNA damage response mechanisms within this organelle. Over the past decades, significant advances have been made in delineating the pathways that safeguard mtDNA integrity. Current evidence indicates that mitochondria rely on a coordinated network of repair, degradation, and quality-control mechanisms to preserve genome stability. Base excision repair (BER) remains the best-characterized and likely the predominant repair pathway. In contrast, bulky and replication-blocking lesions appear to be managed mainly through damage tolerance strategies, including translesion synthesis and repriming, although the molecular framework governing these processes is still not fully defined. Double-strand breaks represent a critical threat to mtDNA stability, and emerging data support the existence of microhomology-mediated end joining (MMEJ)-like and recombination-based pathways in mitochondria, whose regulation and physiological relevance are only beginning to be understood.

Looking forward, a major challenge for the field is to integrate these pathways into a unified model of mitochondrial genome surveillance. It is important to emphasized that the molecular decision process determining whether a damaged mtDNA molecule is repaired, degraded, or eliminated through mitophagy remains poorly understood. A major open question in the field is how mitochondria integrate the extent of genome damage with metabolic status and organelle dynamics to select among these competing quality-control pathways. This unresolved issue is increasingly viewed as a central challenge in understanding mitochondrial genome maintenance. Future studies combining high-resolution imaging, long-read sequencing, targeted mitochondrial genome editing, and quantitative proteomics will be instrumental in clarifying how repair, tolerance, and degradation are coordinated in space and time within the organelle. Moreover, understanding how mtDNA maintenance interfaces with metabolic signaling, innate immunity, and nuclear genome stability will be essential to fully appreciate the systemic consequences of mitochondrial genotoxic stress. Elucidating these mechanisms will not only deepen our knowledge of fundamental mitochondrial biology but may also uncover novel therapeutic opportunities for aging-related disorders, neurodegeneration, cancer, and mitochondrial diseases.

## Data Availability

No data or materials were generated for this article.
